# Improved finite element method for flow, heat and solute transport of Prandtl liquid via heated plate

**DOI:** 10.1038/s41598-022-20332-2

**Published:** 2022-11-16

**Authors:** Muhammad Bilal Hafeez, Marek Krawczuk, Wasim Jamshed, Hajra Kaneez, Syed M. Hussain, El Sayed M. Tag El Din

**Affiliations:** 1 Institute of Mechanics and Machine Design, Faculty of Mechanical Engineering and Ship Technology, Gdansk University of Technology, Narutowicza 11/12, 80-233 Gdańsk, Poland; 2 Department of Mathematics, Capital University of Science and Technology (CUST), Islamabad, 44000 Pakistan; 3 Department of Mathematics, Institute of Space Technology, Islamabad, 44000 Pakistan; 4 Department of Mathematics, Faculty of Science, Islamic University of Madinah, Medina, 42351 Saudi Arabia; 5 Electrical Engineering, Faculty of Engineering and Technology, Future University in Egypt, New Cairo, 11835 Egypt

**Keywords:** Mathematics and computing, Physics

## Abstract

In the current study, a vertical, 3D-heated plate is used to replicate the generation of heat energy and concentration into Prandtl liquid. We discuss how Dufour and Soret theories relate to the equations for concentration and energy. In order to see how effectively particles, interact with heat and a solvent, hybrid nanoparticles are used. It does away with the phenomena of viscous dissipation and changing magnetic fields. The motivation behind the developed study is to optimize solvent and heat storage uses in the biological and industrial domains. This article's major goal is to explore the aspects of thermal energy and mass transfer that influence how nanoparticles, hybrid nanoparticles, and 3D melting surface sheets behave. Variable thermal efficiency and variable mass transfer are combined. The system of generated PDEs (difference equations) includes the concentration, velocity, and heat energy equations. The numerical calculations are done for Silver (Ag), Molybdenum Disulfide (MoS_2_) nanoparticles with Ethylene glycol (C_2_H_6_O_2_) as the base fluid using a boundary layer approach to the mathematical formulation. The system of ODEs is formulated through transformations in order to find a solution. A Galerkin finite element algorithm (G-FEA) is adopted to analyze various aspects versus different parameters. It has been found that motion into hybrid nanoparticles is reduced by motion into nanoparticles. Additionally, differences in heat energy and solvent particle sizes are associated with modifications in magnetic, Dufour, Eckert, and Soret numbers. In contrast to hybrid nanostructures, the output of thermal energy is usually observed to be substantially higher. The magnetic field parameter decreases the particle velocity. In contradiction to the Eckert number, bouncy parameter, and magnetic parameter set values, the maximum quantity of heat energy is obtained. variable thermal conductivity's function. The 3D heated vertical surface convective heat transfer of nanofluids and hybrid nanofluids under the impact of a heat source, thermal radiation, and viscous dissipation has not yet been studied, as far as the authors are aware.

## Introduction

Due to advancements in technology, the synthesis of solid particles of nano-size has become possible. These nanoparticles have been used in many advanced engineering applications. In this sense, transportations of heat, cooling, and thermal systems, engine oil usage, electronic devices, medical sciences, etc. are the sectors where nanofluids (fluid with nanoparticles) play a significant role. The practical direct applications of nanofluids have motivated engineers and scientists to investigate the dynamics of fluids with nanoparticles. Here, let us describe some recent and relevant investigations. For instance, Dogonchi et al.^1^ discussed the simultaneous impact of thermal radiations, thermal relaxation, and dispersion of nanoparticles on heat transfer in fluid over a stretchable surface. Sadeghi et al.^2^ analyzed the role of heat transfer in water enclosures with wavy walls. They also analyzed the impact of internal heat generation on heat transfer enhancement in natural convective flow. Nazir et al.^3^ modeled flow and thermal analysis in hyperbolic tangent liquid inserting hybrid nanostructures past heated plate. They adopted finite element approach to address various aspects. In a solar system that was subjected to the flow of nanoparticles, Zahra et al.^4^ studied the impacts of heat radiations heat flux. Heat transport in fluid with nanoparticles subjected to the magnetic field was explored by Sheikholeslami and Ganji^5^. Using a molecular dynamics technique, Zeeshan and Bhargav^6^ looked at how heat transport in a fluid was affected by dispersion of and in the fluid. Sajjad et al.^7^ investigated how the Darcy-Forchheimer porous medium and nanoparticle affected the transmission of heat and mass in fluid across a flowing fluid.^8–17^ presented the latest updating that involve the traditional nanofluids with the features of heat and mass transmission in a different physical situation. It may be therefore stated that it becomes the universal truth that the effectiveness of thermal conductivity of fluid due to dispersion of a single kind of nanoparticles is lesser than the effectiveness of thermal conductivity of fluid due to the dispersion of hybrid nanoparticles. Therefore, the usage of hybrid nanostructures for optimized thermal enhancement of the working fluid is recommended. Due to this significant reason, several studies on this topic have been conducted. For example, Nazir et al.^18^ studied comparison among hybrid nanoparticles and nanomaterials in base fluid (ethylene glycol) considering Carreau Yasuda martial and thermal properties. By using a non- Fourier theory, Nazir et al.^19^ evaluated the impact of Williamson liquid on the latent heat and density of hybrid nanoparticles that were getting close to thermal decomposition surfaces. In their investigation of the effect of thermal radiation caused by hybrid nanoparticles on fluid between two two plates, Dogonchi et al.^20^ investigated into the efficiency with which the fluid heat was produced. According to a study by Chamkha et al.^21^, magnetic fields, rotating barriers, and hybrid nanoparticles all have an impact on how much heat can be transferred. Masayebidarched et al.^22^ conducted a theoretical investigation for the heat rise in fluid using hybrid nanoparticles. References^23–25^ provide examples of similar publications that discuss the impact of hybrid nanoparticles on heat generation. Many Researchers like^26–32^ did examinations on heat enhancement of nanofluids by blending more than one kind of nanoparticles into base liquid. These examined are engaged to the effects of actual factors, for example, joule heating effect, buoyancy force, and magnetic effect on the heat enhanced of nanofluids. Researchers are recommended to concentrate on these most recent specialists as they likewise caught diverse mathematical impacts, the porosity of the medium, and extending contracting of plates in no-slip effect. Effects of Dufour and Soret were investigated in^33^ under the influence of the solute's mechanism and the thermal properties of a Casson hybrid nanofluid. We observed the improvement in heat transmission caused by nanofluid applications in a car radiator. In^34^ and analysis of considerable thermal energy production in partly ionization of hyperbolic tangent material based on ternary hybrid. For more details see Refs^35–39^.

A thorough review of the literature finds that three-dimensional developing mode models of thermal energy and mass transfer across a heated surface that is expanding vertically while also having hybrid-Prandtl nanofluid present have not yet been addressed. Due to Soret and Dufour effects' inclusion, the mathematical model is developed as being more sophisticated. With a heat source and Joule heating phenomena, a changing magnetic field is introduced. In addition, the hybrid nanofluid has collected variable features in terms of mass diffusion and thermal conductivity. The numerical calculations are done for Silver (Ag), Molybdenum Disulfide (MoS_2_) nanoparticles with Ethylene glycol (C_2_H_6_O_2_) as the base fluid using a boundary layer approach to the mathematical formulation. A finite element simulation is used to develop complex models. Since there are several potential solutions, this new inquiry is divided into five sections. Section "Analysis of flow" presents the problem formulation. Section "Galerkin finite element algorithm: a computational approach" provides an overview of the numerical approach. Section "Results and discussion" of the report discusses the results. This study is concluded in section "Core points and conclusions".

## Analysis of flow

**Figure 1:**
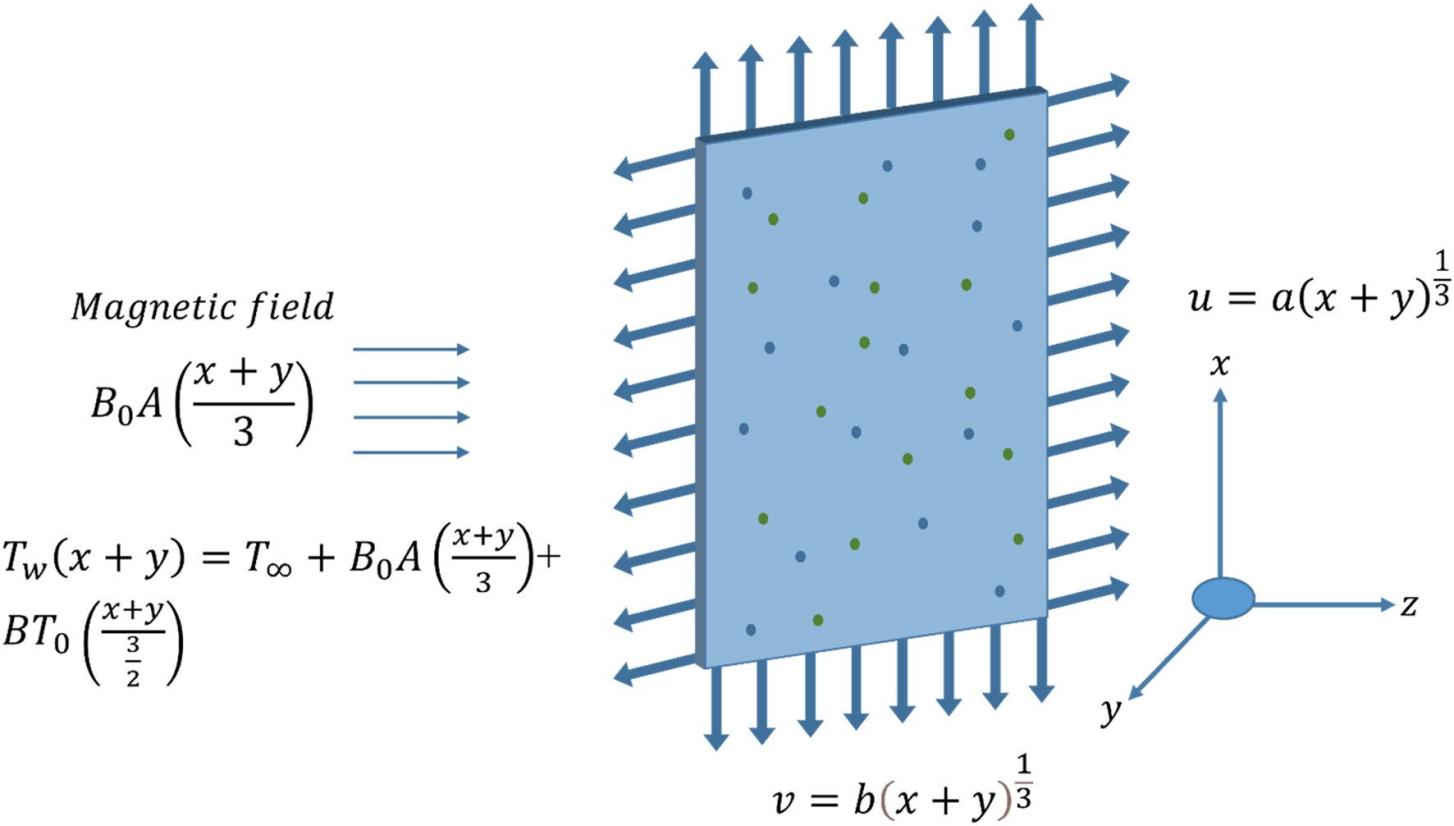
3D vertical surface.

Hybrid nanostructures with properties of heat conduction and solvent molecules in Prandtl liquid are inserted toward a heated area while being influenced by a dynamic magnetic field. A porous surface is used to examine the velocity and heat energy generated by the nanoparticles as well as the effects of Dufour and Soret when temperature variable mass transport and thermal conductivity are present. $Ag$ an is referred to as a nanoparticle, and the composite of $Ag$ and $Cu$ is known as a hybrid nanostructure. Table 1 provides examples of the thermal characteristics of $Ag$ and $Cu$. Figure 1 displays the general concept of the present system. It observed that the magnetic field is inserted along the y-direction, with the x-axis supposed to be in the vertical and the y-axis assumed to be in the horizontal.

**Table 1:** Thermal components of ethylene glycol (C_2_H_6_O_2_), silver (Ag), molybdenum disulfide (MoS_2_).

MoS_2_	Ag	C_2_H_6_O_2_
$\rho =4999$	$\rho =10,490$	$\rho =1113.5$
${C}_{p}=396.20$	${C}_{p}=235$	${C}_{p}=2430$
$k=904.4$	$k=429$	$k=0.253$
$\beta =2.8424\times {10}^{-5}$	$\beta =1.89\times {10}^{-5}$	$\beta =5.8\times {10}^{-4}$
$\sigma =2.09\times {10}^{-5}$	$\sigma =6.30\times {10}^{7}$	$\sigma =4.3\times {10}^{-5}$

**Figure 2:**
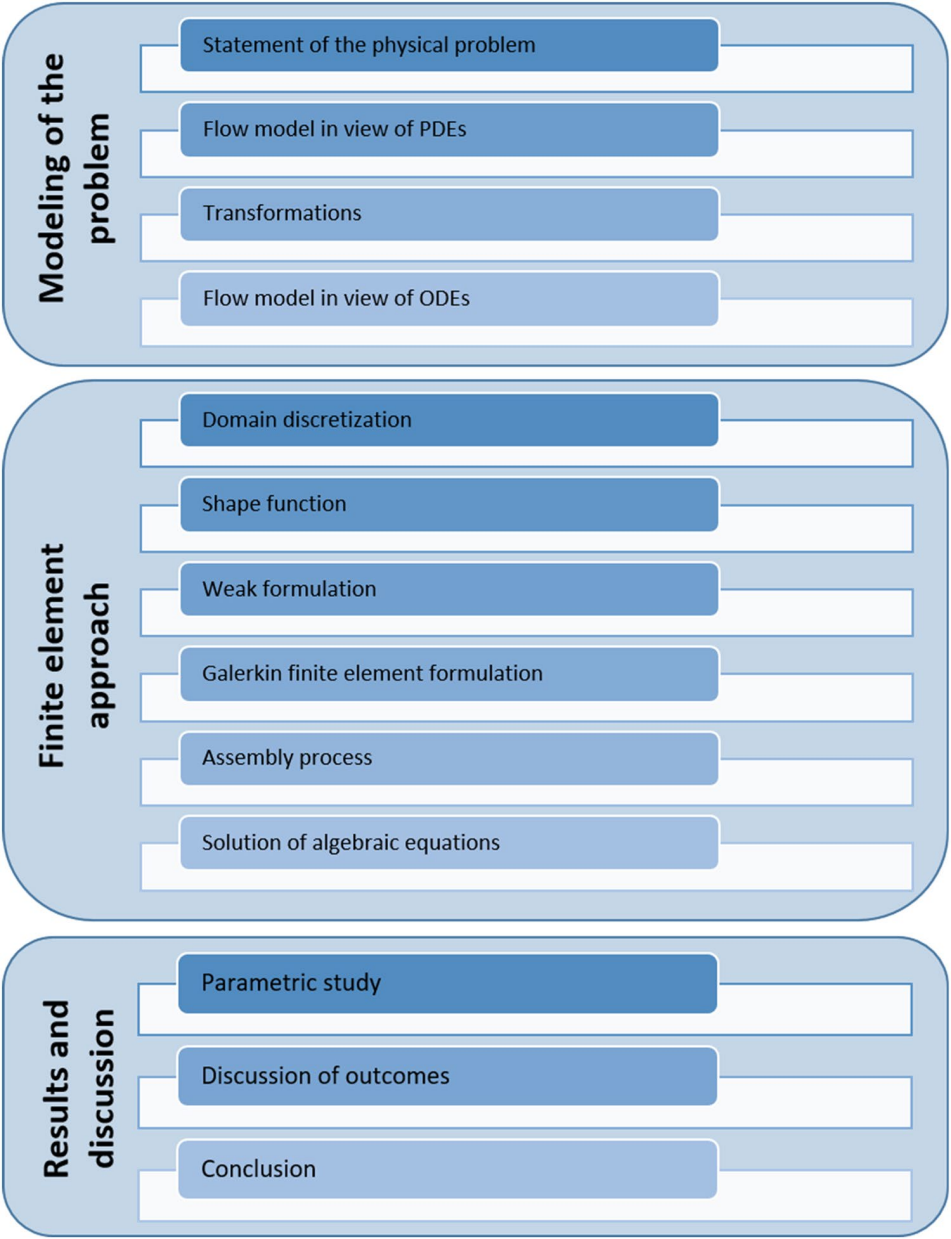
Flow chart of the proposed mathematical model.

Figure 2 displays the schematic chart representation of the mathematical model proposed in this study.

PDEs that characterize the issue include the following^40–42^

1 $$ \frac{\partial u}{{\partial x}} + \frac{\partial v}{{\partial y}} + \frac{\partial w}{{\partial z}} = 0, $$

2 $$ \begin{aligned} & u\frac{\partial u}{{\partial x}} + v\frac{\partial u}{{\partial y}} + w\frac{\partial u}{{\partial z}} = \left( {\beta_{hnf} } \right)_{T} g^{*} \left( {T - T_{\infty } } \right) + \left( {\beta_{hnf} } \right)_{C} g^{*} \left( {C - C_{\infty } } \right) \\ & \quad - \frac{{\sigma_{hnf} }}{{\rho_{hnf} }}B_{o}^{2} A^{2} \left( {x + y} \right)^{{ - \frac{2}{3}}} u - \mu_{hnf} \frac{u}{{K_{1} }} + \nu_{hnf} \left[ {\frac{A}{C}\frac{{\partial^{2} u}}{{\partial z^{2} }} + \frac{A}{{2C^{3} }}\frac{{\partial^{2} u}}{{\partial z^{2} }}\left( {\frac{\partial u}{{\partial z}}} \right)^{2} } \right], \\ \end{aligned} $$

3 $$ \begin{aligned} & u\frac{\partial v}{{\partial x}} + v\frac{\partial v}{{\partial y}} + w\frac{\partial v}{{\partial z}} = \nu_{hnf} \frac{{\partial^{2} v}}{{\partial z^{2} }} + \left( {\beta_{hnf} } \right)_{T} g^{*} \left( {T - T_{\infty } } \right) + \left( {\beta_{hnf} } \right)_{C} g^{*} \left( {C - C_{\infty } } \right) \\ & \quad - \frac{{\sigma_{hnf} }}{{\rho_{hnf} }}B_{o}^{2} A^{2} \left( {x + y} \right)^{{ - \frac{2}{3}}} v - \mu_{hnf} \frac{v}{{K_{1} }} + \nu_{hnf} \left[ {\frac{A}{C}\frac{{\partial^{2} v}}{{\partial z^{2} }} + \frac{A}{{2C^{3} }}\frac{{\partial^{2} v}}{{\partial z^{2} }}\left( {\frac{\partial v}{{\partial z}}} \right)^{2} } \right], \\ \end{aligned} $$

4 $$ \begin{aligned} & u\frac{\partial T}{{\partial x}} + v\frac{\partial T}{{\partial y}} + w\frac{\partial T}{{\partial z}} = \frac{1}{{\left( {\rho c_{p} } \right)_{Thnf} }}\frac{\partial }{\partial z}\left( {K_{Thnf} \left( T \right)\frac{\partial T}{{\partial z}}} \right) \\ & \quad + \frac{{Q_{0} }}{{\left( {\rho c_{p} } \right)_{hnf} }}\left( {T - T_{\infty } } \right) + \frac{{DK_{T} }}{{C_{s} C_{p} }}\frac{{\partial^{2} C}}{{\partial z^{2} }} + \frac{{\sigma_{hnf} B_{o}^{2} A^{2} \left( {x + y} \right)^{{ - \frac{2}{3}}} }}{{\left( {\rho c_{p} } \right)_{hnf} }}\left( {u^{2} + v^{2} } \right), \\ & u\frac{\partial C}{{\partial x}} + v\frac{\partial C}{{\partial y}} + w\frac{\partial C}{{\partial z}} = \frac{\partial }{\partial z}\left( {D_{hnf} \frac{\partial T}{{\partial z}}} \right) + \frac{{D_{T} }}{{T_{\infty } }}\frac{{\partial^{2} T}}{{\partial z^{2} }}, \\ \end{aligned} $$

System of Eqs. (1)–(4) BCs are^43,44^

5 $$ \begin{aligned} u & = U_{w} \left( { = a\left( {x + y} \right)^{\frac{1}{3}} } \right),v = V_{w} \left( { = b\left( {x + y} \right)^{\frac{1}{3}} } \right), w = 0 \\ T & = T_{w} \left( { = cT_{o} \left( {x + y} \right)^{\frac{2}{3}} + T_{\infty } } \right),\quad C = C_{w} \left( { = dC_{o} \left( {x + y} \right)^{\frac{2}{3}} + C_{\infty } } \right)\quad as\quad y = 0 \\ u & = 0, v = 0,T \to T_{\infty } ,C \to C_{\infty } \quad as\quad y \to \infty \\ \end{aligned} $$

Correlations among hybrid nanostructures and nanomaterial in ethylene glycol are ^43^

6 $$ \begin{aligned} \rho_{hnf} & = \left[ {\left( {1 - \phi_{2} } \right)\left\{ {\left( {1 - \phi_{1} } \right)\rho_{f} + \phi_{1} \rho_{s1} } \right\}} \right] + \phi_{2} \rho_{s2} ,\rho_{nf} = \left( {1 - \phi } \right)\rho_{f} + \phi \rho_{s} \\ \left( {\rho C_{p} } \right)_{nf} & = \left( {1 - \phi } \right)\left( {\rho C_{p} } \right)_{f} + \phi \left( {\rho C_{p} } \right)_{s} , \\ \left( {\rho C_{p} } \right)_{hnf} & = \left[ {\left( {1 - \phi_{2} } \right)\left\{ {\left( {1 - \phi_{1} } \right)\left( {\rho C_{p} } \right)_{f} + \phi_{1} \left( {\rho C_{p} } \right)_{s1} } \right\}} \right] + \phi_{1} \left( {\rho C_{p} } \right)_{s2} \\ \end{aligned} $$

7 $$ \begin{aligned} \frac{{k_{nf} }}{{k_{f} }} & = \left\{ {\frac{{k_{s} + \left( {n + 1} \right)k_{f} - \left( {n - 1} \right)\phi \left( {k_{f} - k_{s} } \right)}}{{k_{s} + \left( {n - 1} \right)k_{f} + \phi \left( {k_{f} - k_{s} } \right)}}} \right\} , \quad \mu_{nf} = \frac{{\mu_{f} }}{{\left( {1 - \phi } \right)^{2.5} }} \\ \mu_{hnf} & = \frac{{\mu_{f} }}{{\left( {1 - \phi_{2} } \right)^{2.5} \left( {1 - \phi_{1} } \right)^{2.5} }}, \; - \frac{{\sigma_{hnf} }}{{\sigma_{f} }} = \left( {1 + \frac{{3\left( {\sigma - 1} \right)\phi }}{{\left( {\sigma + 2} \right) - \left( {\sigma - 1} \right)\phi }}} \right) \\ \end{aligned} $$

8 $$ \begin{aligned} \frac{{k_{hnf} }}{{k_{bf} }} & = \left\{ {\frac{{k_{s2} + \left( {n - 1} \right)k_{bf} - \left( {n - 1} \right)\phi_{2} \left( {k_{bf} - k_{s2} } \right)}}{{k_{s2} + \left( {n - 1} \right)k_{bf} - \phi_{2} \left( {k_{bf} - k_{s2} } \right)}}} \right\} \\ \frac{{\sigma_{hnf} }}{{\sigma_{f} }} & = \left( {\frac{{\sigma_{s2} + 2\sigma_{f} - 2\phi_{2} \left( {\sigma_{bf} - \sigma_{s2} } \right)}}{{\sigma_{s2} + 2\sigma_{f} + \phi_{2} \left( {\sigma_{bf} - \sigma_{s2} } \right)}}} \right) \\ \end{aligned} $$

Thermal conductivity and mass diffusion based on temperature are defined as^43^

9 $$ K_{hnf} \left( T \right) = K_{hnf} \left( {1 + \varepsilon_{1} \frac{{T - T_{\infty } }}{{T_{w} - T_{\infty } }}} \right), \quad D_{hnf} \left( T \right) = K_{hnf} \left( {1 + \varepsilon_{2} \frac{{T - T_{\infty } }}{{T_{w} - T_{\infty } }}} \right), $$

Next, the similarity transformation is^40^

10 $$ \begin{aligned} u & = a\left( {x + y} \right)^{\frac{1}{3}} ,\;v = a\left( {x + y} \right)^{\frac{1}{3}} , \;\eta = \sqrt {\frac{a}{{\nu_{f} }}} \left( {x + y} \right)^{{ - \frac{1}{3}}} z, \\ w & = - \sqrt {a\nu_{f} } \left( {x + y} \right)^{{ - \frac{1}{3}}} \left( {\frac{2}{3}\left( {f + g} \right) - \frac{1}{3}\eta \left( {f^{\prime } + g^{\prime } } \right)} \right),\quad \theta = \frac{{T - T_{\infty } }}{{T_{w} - T_{\infty } }},\quad \phi = \frac{{C - C_{\infty } }}{{C_{w} - C_{\infty } }} \\ \end{aligned} $$

In Eqs. (1)–(5), similarity transformation is used, we have

11 $$ \begin{aligned} & \frac{{\nu_{hnf} }}{{v_{f} }}\left( {\alpha_{1} f^{\prime \prime \prime } + \alpha_{2} f^{\prime \prime 2} f^{\prime \prime \prime } } \right) - \frac{1}{3}\left( {f^{\prime } + g^{\prime } } \right)f^{\prime } + \frac{2}{3}\left( {f + g} \right)f^{\prime \prime } + \left( {Gr} \right)_{t} \theta \\ & \quad + \left( {Gr} \right)_{c} \phi - \left( {\frac{{\sigma_{hnf} }}{{\sigma_{f} }}} \right)\left( {\frac{{\rho_{f} }}{{\rho_{hnf} }}} \right)Mf^{\prime } - \left( {\frac{{\mu_{hnf} }}{{\mu_{f} }}} \right)K^{*} f^{\prime } = 0 \\ &f^{\prime } \left( 0 \right) = 1, \;f\left( 0 \right) = 0, \; f^{\prime } \left( \infty \right) \to 0, \\ \end{aligned} $$

12 $$ \begin{aligned} & \frac{{\nu_{hnf} }}{{v_{f} }}\left( {\alpha_{1} g^{\prime \prime \prime } + \alpha_{2} g^{\prime \prime 2} g^{\prime \prime } } \right) - \frac{1}{3}\left( {f^{\prime } + g^{\prime } } \right)g^{\prime } + \frac{2}{3}\left( {f + g} \right)g^{\prime \prime } + \left( {Gr} \right)_{t} \theta \\ & \quad + \left( {Gr} \right)_{c} \phi - \left( {\frac{{\sigma_{hnf} }}{{\sigma_{f} }}} \right)\left( {\frac{{\rho_{f} }}{{\rho_{hnf} }}} \right)Mg^{\prime } - \left( {\frac{{\mu_{hnf} }}{{\mu_{f} }}} \right)K^{*} g^{\prime } = 0 \\ & g^{\prime } \left( 0 \right) = \beta , \;g\left( 0 \right) = 0, \; g^{\prime } \left( \infty \right) \to 0, \\ \end{aligned} $$

13 $$ \begin{aligned} & \frac{{K_{hnf} }}{{K_{f} }}\left[ {\left( {1 + \varepsilon_{1} \theta } \right)\theta^{\prime \prime } + \varepsilon_{1} \left( {\theta^{\prime } } \right)^{2} } \right] + \left( {\frac{{\left( {\rho c_{p} } \right)_{hnf} }}{{\left( {\rho c_{p} } \right)_{f} }}} \right)\frac{2}{3}Pr\left( {f + g} \right)\theta^{\prime } - \left( {\frac{{\left( {\rho c_{p} } \right)_{hnf} }}{{\left( {\rho c_{p} } \right)_{f} }}} \right)\frac{2}{3}Pr\left( {f^{\prime } + g^{\prime } } \right)\theta \\ & - Pr\beta^{*} \theta + \left( {\frac{{\left( {\rho c_{p} } \right)_{hnf} }}{{\left( {\rho c_{p} } \right)_{f} }}} \right)DuPr\phi^{\prime \prime } + \left( {\frac{{\sigma_{hnf} }}{{\sigma_{f} }}} \right)MPrEc\left( {f^{\prime } + g^{\prime } } \right)^{2} = 0 \\ & \quad \theta \left( 0 \right) = 1, \;\theta \left( \infty \right) \to 0, \\ \end{aligned} $$

14 $$ \begin{aligned} & \frac{{D_{hnf} }}{{D_{f} }}\left[ {\left( {1 + \varepsilon_{1} \varphi } \right)\varphi^{\prime \prime } + \varepsilon_{2} \varphi^{\prime } \theta^{\prime } } \right] + \frac{2}{3}Sc\left( {f + g} \right)\phi^{\prime } - \frac{2}{3}Sc\left( {f^{\prime } + g^{\prime } } \right)\phi + SrSc\theta^{\prime \prime } = 0 \\ & \phi \left( 0 \right) = 1, \;\phi \left( \infty \right) \to 0, \\ \end{aligned} $$

The dimensionless numbers and defined here

15 $$ \begin{aligned} \left( {Gr} \right)_{t} & = \frac{{\left( {\beta_{hnf} } \right)_{T} g^{*} cT_{0} }}{{a^{2} }} ,\left( {Gr} \right)_{c} = \frac{{\left( {\beta_{hnf} } \right)_{C} g^{*} dC_{0} }}{{a^{2} }}, M = \frac{{\sigma_{f} }}{{\rho_{f} }}\frac{{B_{0}^{2} A^{2} }}{a}, K^{*} = \frac{{\mu_{f} }}{{ak_{1} }}, \\ Ec & = \frac{1}{{(c_{p} )_{f} }}\frac{{a^{2} }}{{cT_{0} }} , \; \beta^{*} = \frac{{Q_{0} }}{{a\left( {\rho c_{p} } \right)_{f} }} , \;Du = \frac{{DK_{T} dC_{0} }}{{C_{s} C_{p} V_{f} cT_{0} }}, \;Sc = \frac{{V_{f} }}{{d_{f} }} ,\;Sr = \frac{{D_{T} T_{0} }}{{(T_{\infty } C_{0} )V_{f} }} . \\ \end{aligned} $$

Table 1 describes the set of parameters that have been used in this investigation for practical purposes^43,44^.

Surface-based forces are described as

16 $$ C_{fx} = \frac{{\left. {\frac{\partial u}{{\partial z}}} \right|_{z = 0} }}{{\rho_{f} \left( {U_{w} } \right)^{2} }} = \frac{{\left( {1 - \phi_{1} } \right)^{ - 2.5} }}{{\left( {1 - \phi_{2} } \right)^{2.5} \left( {Re} \right)^{1.5} }}\left[ {\alpha_{1} f^{\prime \prime } \left( 0 \right) + \alpha_{2} \left( {f^{\prime \prime \prime } \left( 0 \right)} \right)^{3} } \right], $$

17 $$ C_{gy} = \frac{{\left. {\frac{\partial v}{{\partial z}}} \right|_{z = 0} }}{{\rho_{f} \left( {U_{w} } \right)^{2} }} = \frac{{\left( {1 - \phi_{1} } \right)^{ - 2.5} }}{{\left( {1 - \phi_{2} } \right)^{ - 2.5} \left( {Re} \right)^{1.5} }}\left[ {\alpha_{1} g^{\prime \prime } \left( 0 \right) + \alpha_{2} \left( {g^{\prime \prime \prime } \left( 0 \right)} \right)^{3} } \right]. $$

Nusselt number is

18 $$ Nu = - \frac{{\left. {\left( {x + y} \right)K_{hnf} \frac{\partial T}{{\partial y}}} \right|_{y = 0} }}{{k_{f} \left( {T - T_{\infty } } \right)}} = - \frac{{K_{hnf} }}{{k_{f} \left( {Re} \right)^{1.5} }}\theta^{\prime } \left( 0 \right), $$

the mass flux is

19 $$ Sh = \frac{{\left. {\left( {x + y} \right)D_{hnf} \frac{\partial C}{{\partial y}}} \right|_{y = 0} }}{{D_{f} \left( {C - C_{\infty } } \right)}} = - \frac{{D_{hnf} }}{{D_{f} \left( {Re} \right)^{1.5} }}\phi^{\prime } \left( 0 \right), $$

where $Re = \frac{{xU_{w} }}{{\nu_{f} }}$, the Reynolds number.

## Galerkin finite element algorithm: a computational approach

The provided problem is solved using the Galerkin finite element algorithm (G-FEA). The FEMs explain the method are listed here^45–49^. Some limitations on finite element method are listed below.

- Analysis of finite elements is perceived as more complex in view of understanding rather than others numerical methods;
- Finite element method can be expensive in term of computational cost as compared to other methods;
- Large data is needed for mesh free analysis.
- Construction of the residual equations is done.
- The residual is integrated across a conventional discrete time domain component.
- Stiffness matrices are generated after calculating the weighted residual integrals using by G-FEM technique.
- By following the restrictions of element assembly, the nonlinear equations are modeled. Under the constraints for calculation, the linearized system is solved $10^{ - 3}$.
- Results are obtained that are grid independent after the convergence is validated. It utilizes the error analysis criterion.
  20 $$ \left| {\frac{{\eta^{i + 1} - \eta^{i} }}{{\eta^{i} }}} \right| < 10^{ - 5} . $$

Examples of the parametric research are provided to demonstrate the effects of heat generation, porous media, mass diffusion, thermal diffusivity, the rate of heat flow and mass diffusion on the study of thermal energy and mass transfer in 3D Newtonian fluid flow. Table 2 shows 300 element mesh-free issue analysis results.

**Table 2:** Shows a study of temperature, velocities, and concentrations using 300 elements of a grid.

No. of elements	$f{^{\prime}}\left(\frac{{\eta }_{max}}{2}\right)$	$g{^{\prime}}\left(\frac{{\eta }_{max}}{2}\right)$	$\theta \left(\frac{{\eta }_{max}}{2}\right)$	$\phi \left(\frac{{\eta }_{max}}{2}\right)$
30	0.7840956617	0.003662478537	0.0036624785	0.00010687187
60	0.8208393123	0.09000953164	0.1110267939	0.00506883934
90	0.8299235799	0.002650512986	0.01342155803	0.00005691660
120	0.6909729670	0.0004285360556	0.01039153713	0.04544824477
150	0.6949838844	0.0004160495348	0.01033124362	0.04500018680
180	0.6979030185	0.0004100790678	0.01029442761	0.04477827150
210	0.7002801242	0.0004086029651	0.01027192133	0.04470747392
240	0.7023798401	0.0004105345687	0.01025902553	0.04474784442
270	0.7043301851	0.0004151139823	0.01025290562	0.04487205678
300	0.7061806472	0.0004216405675	0.01025152760	0.04505585937

## Results and discussion

To investigate the physics of the issue described in the previous part, parametric research has been presented. The fractionated finite element method is used to generate a numerical solution. Using FEM, the mathematical model for mass and thermal energy transfer in non-Newtonian flows beyond a surface with thermal and wall density gradients is numerically solved.

**Figure 3:**
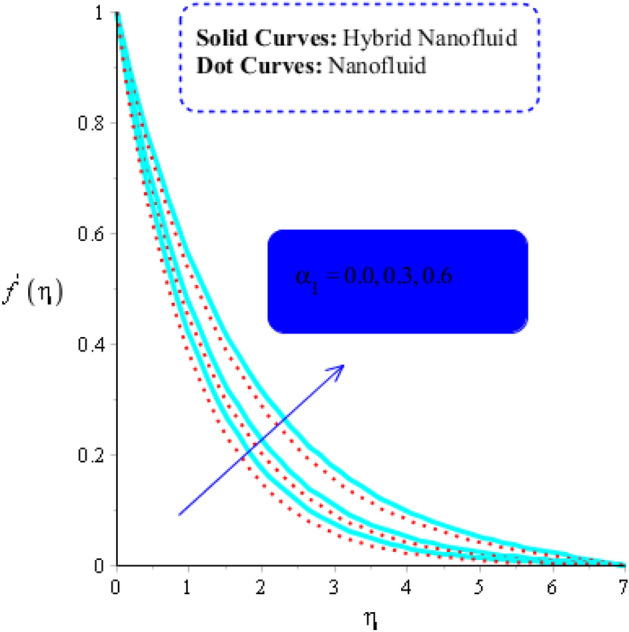
Influence of $\alpha_{1}$ on $f^{\prime }$ when $\left( {Gr} \right)_{t} = 0.7,\; Pr = 3, \;Sc = 0.6, \;K^{*} = 0.5, \;Ec = 0.01,\;\left( {Gr} \right)_{c} = 0.5,\;M = 0.2, \;\beta^{*} = 0.2, \;Sr = 0.7,$ and $Du = 0.2, \;Du = 0.2, \;\varepsilon_{1} = 0.4,\;\varepsilon_{2} = 0.5, \;\alpha_{2} = 5.0.$

**Figure 4:**
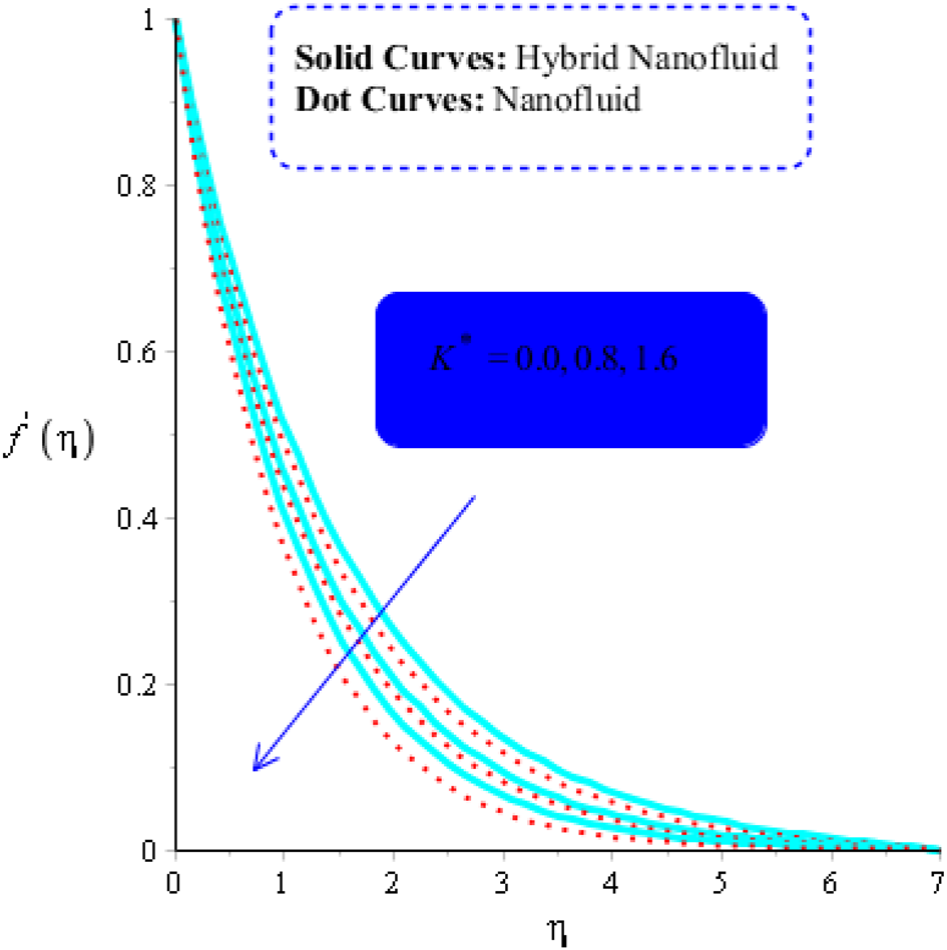
Impact of *K* ∗ on *f*′ when $Gr$ *t* = 0.5, *Pr* = 4, *Sc* = 5, β = 0.2, *Ec* = 0.001,$Gr$ *c* = 0.3, *M* = 0.5, β ∗  = 0.2, *Sr* = 0.1 and *Du* = 0.2, ϵ1 = 0.7, ϵ2 = 0.5, α1 = 0.5, α2 = 5.0.

As yield stress is the property that prevents fluid from deforming until a specific applied stress is reached. The fluid must oppose the applied tension in order to reach the equilibrium condition, the yield stress must increase. As a result, a drop in the velocity profile (in both $x$ and $y$ -components) is seen (see Figs. 3 and 4). Figures 3 and 6 have indeed been produced to illustrate how fluid parameters affect velocity curves. It is noticed that fluid becomes thin versus the higher impacts of fluid parameter.

**Figure 5:**
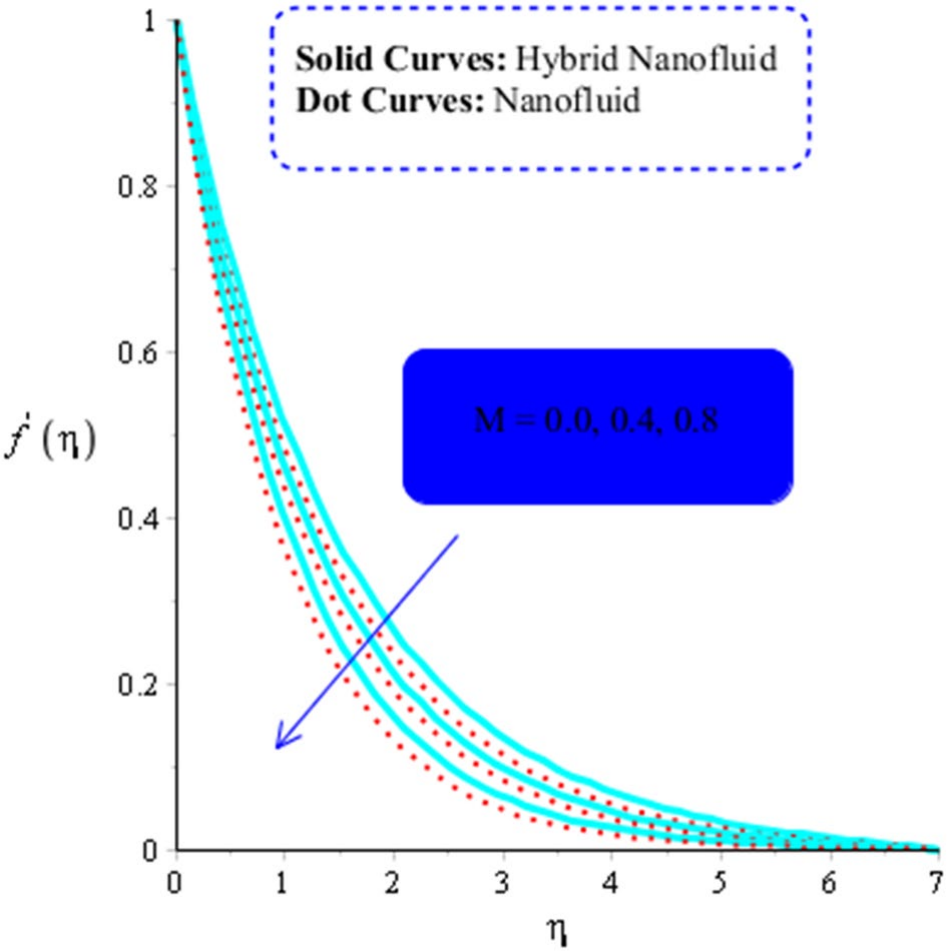
Impact of $M$ on $f^{\prime}$ , $\left( {Gr} \right)_{t} = 0.7, Pr = 8, Sc = 5, \beta = 0.2, Ec = 0.1,\left( {Gr} \right)_{c} = 0.5,K^{*} { } = 0.1,\beta^{*} = 0.2, Sr = 0.1,$ and $Du = 0.2, \;\varepsilon_{1} = 0.3, \;\varepsilon_{2} = 0.7, \;\alpha_{1} = 0.5, \;\alpha_{2} = 3.0.$

**Figure 6:**
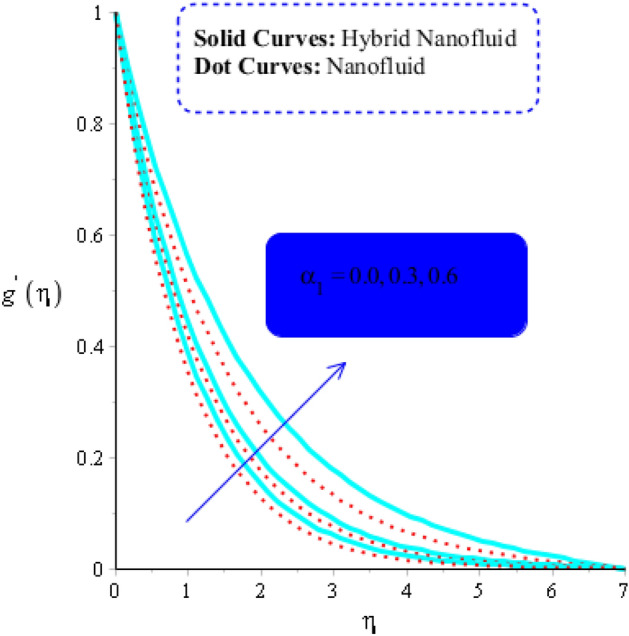
Impact of $\alpha_{1}$ on $g^{\prime}$ when $\left( {Gr} \right)_{t} = 0.5, \;Pr = 5, \;Sc = 5, \;K^{*} = 0.1, \;Ec = 0.001,\left( {Gr} \right)_{c} = 0.7,\;M = 0.5,\;\beta^{*} = 0.2, \;Sr = 0.1,$ and $Du = 0.2, \;Du = 0.2, \;\varepsilon_{1} = 0.3, \;\varepsilon_{2} = 0.5, \;\alpha_{2} = 3.0.$

**Figure 7:**
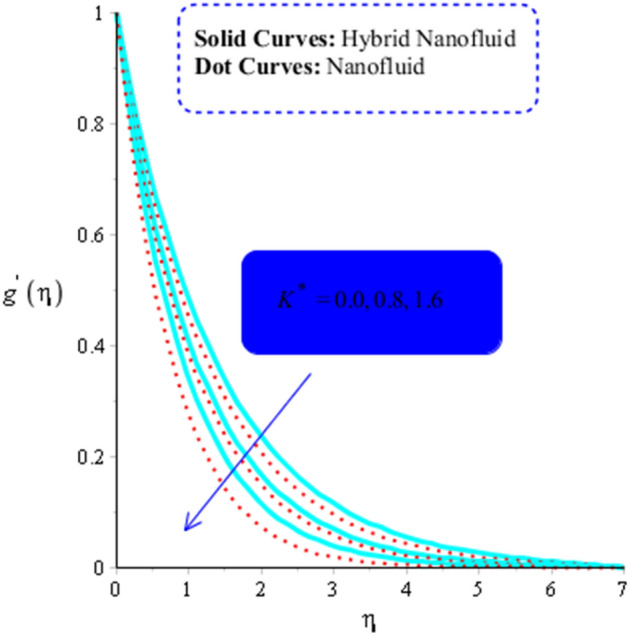
Impact of $K^{*}$ on $g^{\prime}$ when $\left( {Gr} \right)_{t} = 0.5, \;Pr = 5, \;Sc = 5, \;\beta = 0.2, \;Ec = 3,\;\left( {Gr} \right)_{c} = 0.3,\;M = 0.5,\;\beta^{*} = 0.2, \;Sr = 0.1, \;Du = 0.8, \;\varepsilon_{1} = 0.3,\; \varepsilon_{2} = 0.3, \;\alpha_{1} = 0.5, \alpha_{2} = 3.0.$

**Figure 8:**
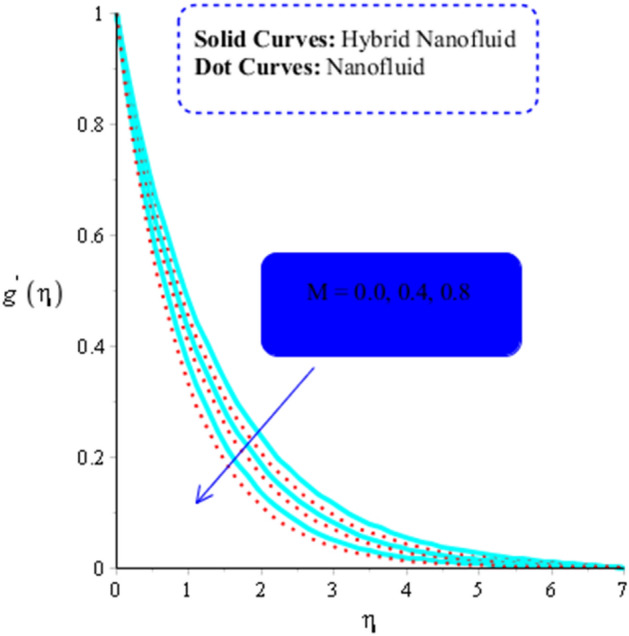
Impact of $M$ on $g^{\prime }$, $\left( {Gr} \right)_{t} = 0.5, Pr = 4, Sc = 5, \beta = 0.2, Ec = 0.001,\left( {Gr} \right)_{c} = 0.3,K^{*} { } = 0.1,\beta^{*} = 0.2, Sr = 0.1,$ and $Du = 0.2, Du = 0.2,\; \varepsilon_{1} = 0.3, \;\varepsilon_{2} = 0.5,\; \alpha_{1} = 0.5,\; \alpha_{2} = 3.0.$

The numerous numerical experiments are run using various samples of customizable elements. The numerical experiments yield a few significant findings. It is significant to notice that solid curves are concerned with flow, heat exchange, and mass transfer in hybrid nanofluid, whereas dashed curves are connected with flows, heat exchange, and mass transfer in MoS_2_-Ag-hybrid nanofluid. Consequently, the flow in both the $x$- and $y$ directions slow down (see Figs. 5 and 6). Moreover, Figs. 7 and 8 shows the parameter $k^{*}$ related to the resistance of a porous media to fluid flow and how it affects how fluid particles move. These figures likewise show declining velocities. Additionally, these figures demonstrate that compared to mono nano-Casson fluid, hybrid nano-Casson fluid encounters greater resistance from the porous media. When compared to hybrid nano-Casson fluid, the mono, nano-Casson fluid has a wider viscosity region.

### Fluid flow versus the magnetic field's function

The magnetic field and the Lorentz force are directly related. The evolution of $M$ can be used to calculate the Lorentz force's influence on flow. The adverse impact of the Lorentz force increases with increasing values of $M$. As a result, the Lorentz force causes flow to slow down. (See Figs. 7 and 8). As a result, change in the magnetic field is used to reduce boundary layer thickness (the intensity of applied). The Lorentz force for the flow of MoS_2_-Ag-hybrid nanofluid is also reported to be greater than the Lorentz force for the flow of MoS_2_-nanofluid.

### Temperature field in relation to changes in key model parameters

**Figure 9:**
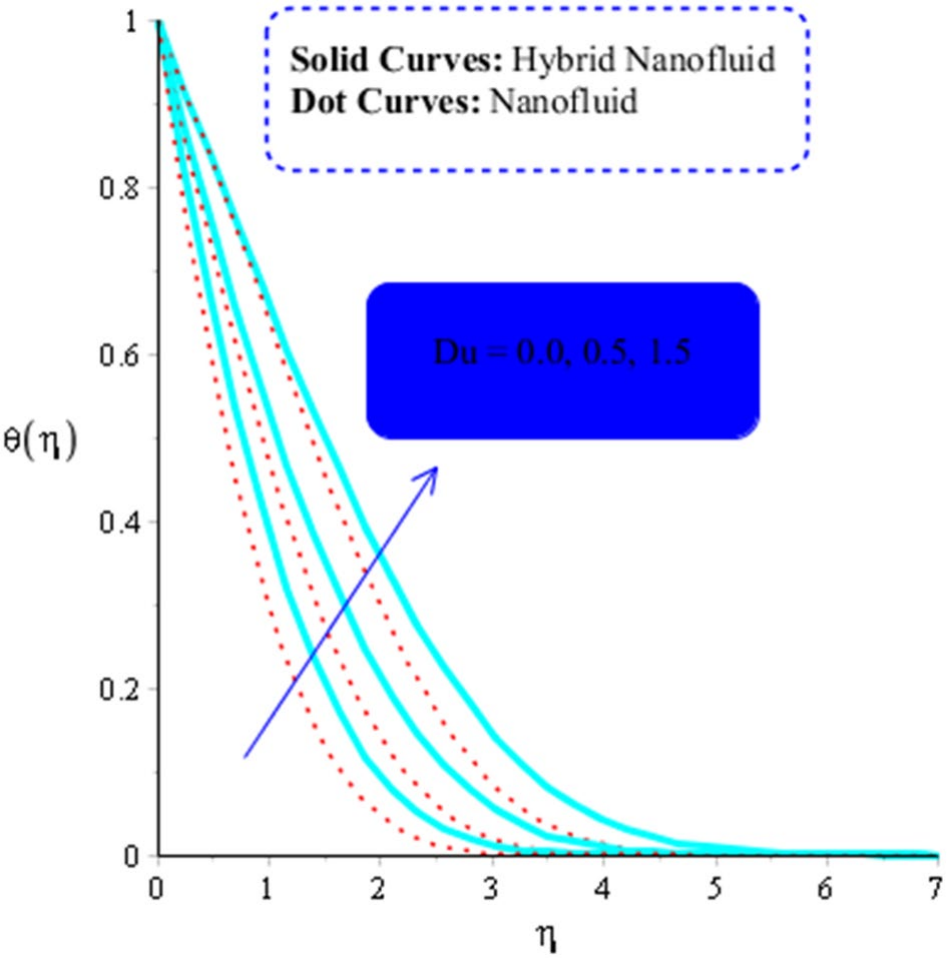
Influence of $Du$ on $\theta$ when $\left( {Gr} \right)_{t} = 5, \;Pr = 7, \;Sc = 0.4, \;\beta = 0.2, \;Ec = 0.001,\;\left( {Gr} \right)_{c} = 0.3,K^{*}  = 0.1,\;\beta^{*} = 0.2,\; Sr = 0.1, \;M = 0.5, \;Du = 0.2, \;\varepsilon_{1} = 0.3, \;\varepsilon_{2} = 0.5,\; \alpha_{1} = 0.5, \;\alpha_{2} = 3.0.$

For both MoS_2_ and Ag nanofluid, the effects of $M$, $Ec$ $Pr$,$\beta^{*}$, and $Du, \left( {Gr} \right)_{t}$, versus thermal energy are studied. Figures 9 through 13 demonstrate the observed influence of these parameters, accordingly. The Dufour number refers to the input variable $Du$. When transcript of heat energy resulting from gradient of concentration is taken into account it shows in the non—dimensional the energy equation's form. The heat transport is examined due to compositional variations brought on by nanoparticles and soluble compounds distributed throughout the fluid. Figure 9 illustrates how $Du$ affects the temperature of MoS_2_-nanofluid and MoS_2_-Ag-hybrid nanofluid. As a factor of $Du$, the temperature of both types of fluids tends to rise.

**Figure 10:**
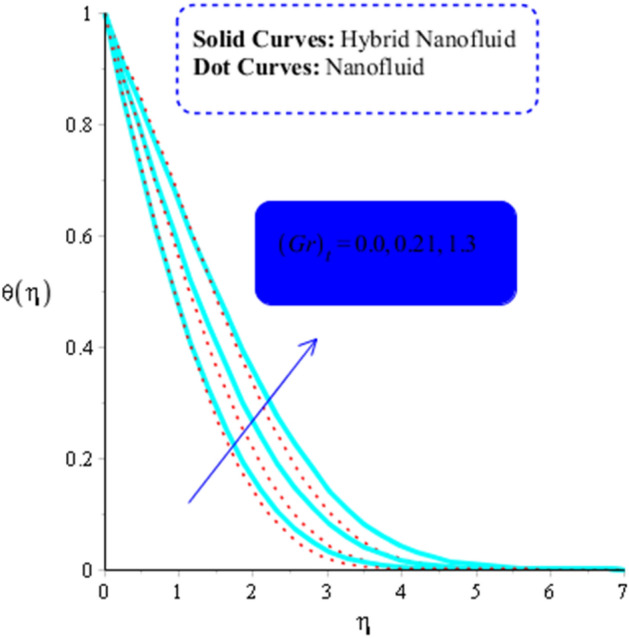
Impact of $\left( {Gr} \right)_{t}$ on $\theta$ when $Du = 0.3, \;Pr = 4, \;Sc = 5, \beta = 0.7, \;Ec = 0.01,\;\left( {Gr} \right)_{c} = 0.3,\;K^{*} { } = 0.1,\;\beta^{*} = 0.2, \;Sr = 0.1, \;M = 0.5, \;Du = 0.2, \;\varepsilon_{1} = 0.3,\;\varepsilon_{2} = 0.5,\; \alpha_{1} = 0.7,\; \alpha_{2} = 3.0.$

**Figure 11:**
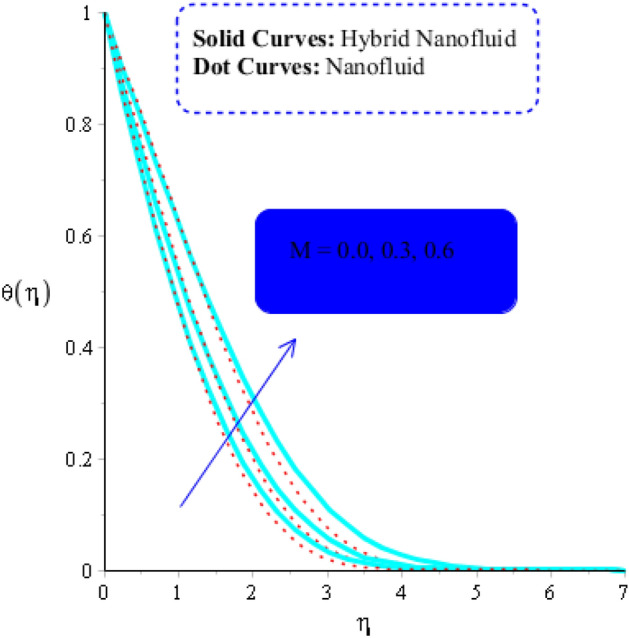
Impact of $M$ on $\theta$ when $Du = 0.2, \;Pr = 4, \;Sc = 5, \beta = 0.2, Ec = 0.001,\left( {Gr} \right)_{c} = 0.3,\;K^{*} { } = 0.1,\;\beta^{*} = 0.2, \;Sr = 0.1, \left( {Gr} \right)_{t} = 0.5, \;Du = 0.2,\; \varepsilon_{1} = 0.3, \;\varepsilon_{2} = 0.5, \;\alpha_{1} = 0.5, \;\alpha_{2} = 3.0.$

**Figure 12:**
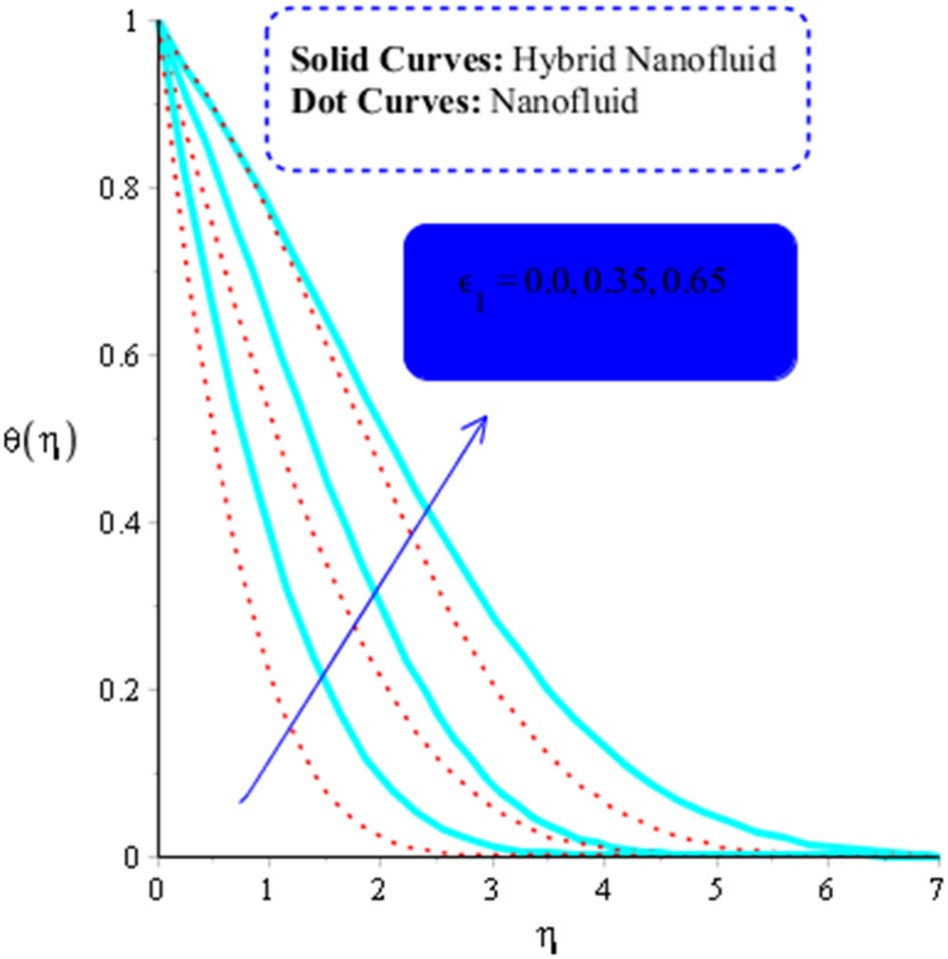
Influence of $\varepsilon_{1}$ on $\theta$ when $Du = 0.2, \;M = 0.5, \;Sc = 5, \;\beta = 0.2, \;Ec = 0.001,\left( {Gr} \right)_{c} = 0.3,K^{*} { } = 0.1,\;Pr = 4, \;Sr = 0.1, \; \left( {Gr} \right)_{t} = 0.5, \;Du = 0.2, \;\varepsilon_{2} = 0.5, \;\alpha_{1} = 0.5, \;\alpha_{2} = 3.0.$

**Figure 13:**
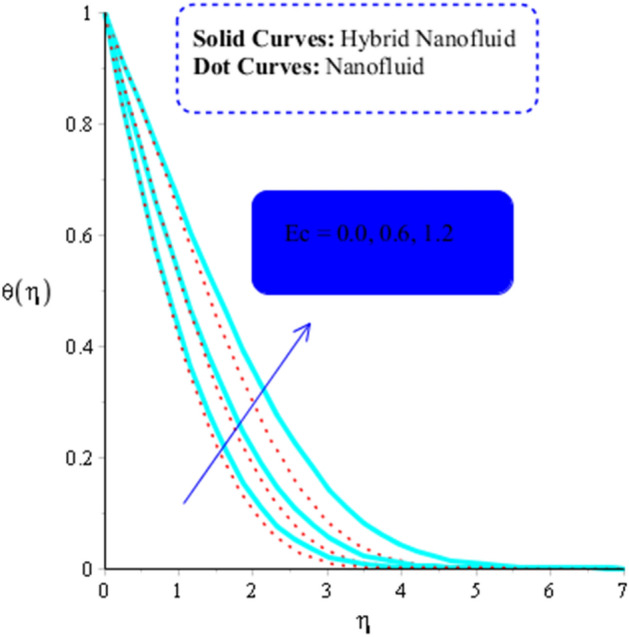
Influence of $Ec$ on $\theta$ when $Du = 0.1, M = 0.2, Sc = 5, Pr = 4,\left( {Gr} \right)_{c} = 0.3,K^{*} { } = 0.1,\beta^{*} = 0.2, Sr = 0.1, \left( {Gr} \right)_{t} = 0.5, Du = 0.2,\; \varepsilon_{1} = 0.3,\; \varepsilon_{2} = 0.5, \alpha_{1} = 0.5, \alpha_{2} = 3.0.$

As a function of $Du$, the temperature of the both types of fluids tend to rise. $Du$ has less of an impact on the temperature of MoS_2_-nanofluid than it does on the temperature of MoS_2_-Ag-hybrid nanofluid. Figure 10 depicts the effects of fluid particles on the temperature of MoS_2_-Ag-hybrid nanofluid. When the flow is enhanced by a positive drag force, the situation is $\left( {Gr} \right)_{ \in } > 0$. If buoyancy force is negative, however, as it is in the situation in $\left( {Gr} \right)_{t} < 0$, the flow is referred to as opposed flow. The Heat and mass transfer effect occurs when heat is produced during conversion and is added to a medium, such as fluid. Consequently, Fig. 11 displays the temperature as a result of Joule heating. Additionally, it is found that the hybrid nanofluid exhibits a stronger Joule heating phenomena than the MoS_2_ does (mono-fluid). Additionally, the parameter $\beta^{*}$ arises as a result of the energy equation's energy equation's heat generation part not being dimensioned. The fluid absorbs the heat that is produced, which raises the fluid's temperature. Figure 12 provides evidence to support this observation. The temperature of fluids considerably increases as a result of fluid motion (nanofluid and MoS_2_-Ag-hybrid nanofluid). Simulations reveal that the fluid velocity in the MoS_2_ is larger than that in the MoS_2_-Ag-hybrid nanofluid. These findings are evident from Fig. 13.

### Role of mass diffusion

**Figure 14:**
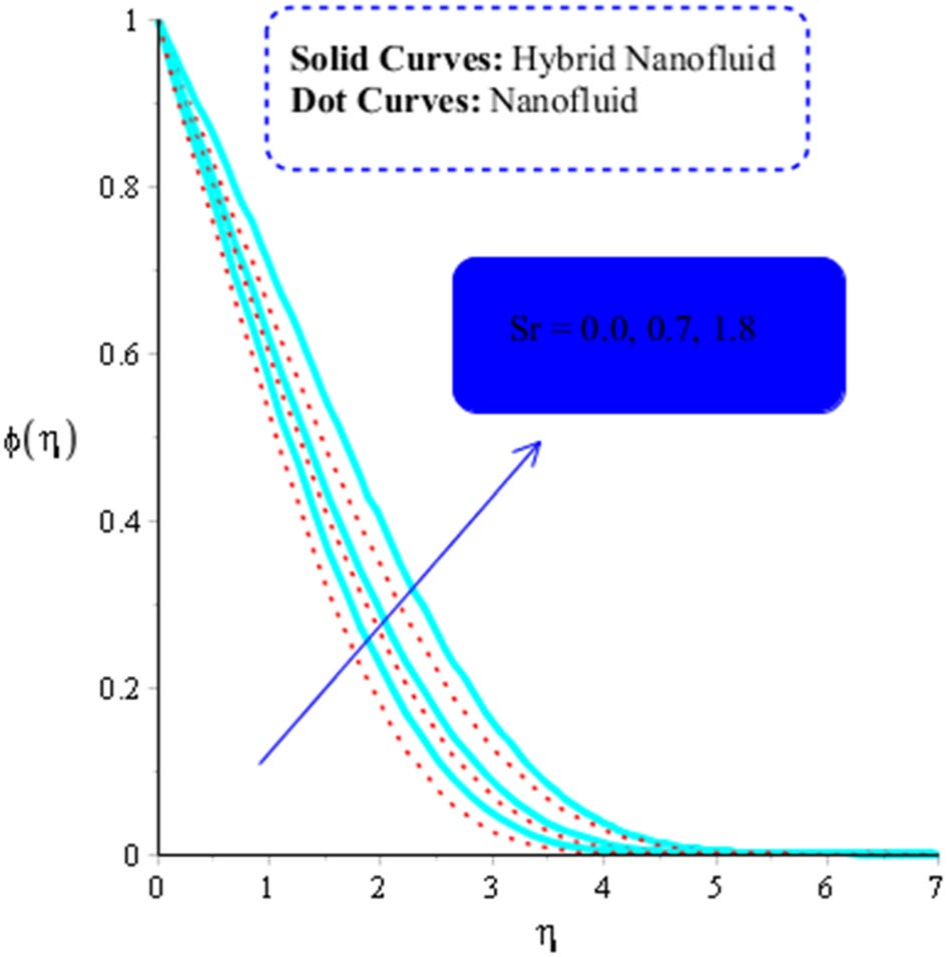
Influence of $Sr$ on $\phi$ when $Du = 0.2, M = 0.5, Sc = 5, Pr = 4,\left( {Gr} \right)_{c} = 0.3,K^{*} { } = 0.1,\beta^{*} = 0.2, Ec = 0.001,$ and $\left( {Gr} \right)_{t} = 0.5, Du = 0.2,\; \varepsilon_{1} = 0.3,\; \varepsilon_{2} = 0.5, \alpha_{1} = 0.5, \alpha_{2} = 3.0.$

**Figure 15:**
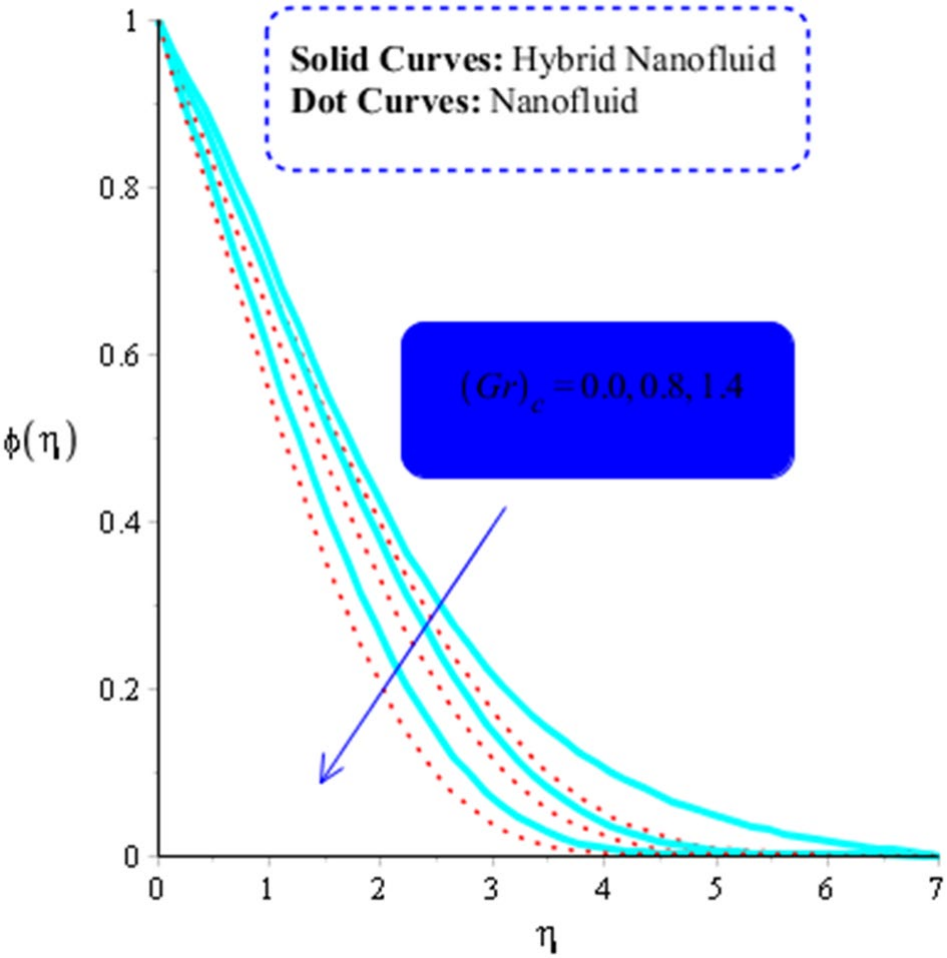
Influence of $\left( {Gr} \right)_{c}$ on $\phi$ when $Du = 0.4, M = 0.6, Sr = 0.1, Pr = 5,Sc = 0.03,$ $K^{*} { } = 0.1,\beta^{*} = 0.2, Ec = 0.001,$ and $\left( {Gr} \right)_{t} = 0.5, Du = 0.2,\;\varepsilon_{1} = 0.3, \;\varepsilon_{2} = 0.5, \alpha_{1} = 0.5, \alpha_{2} = 3.0.$

**Figure 16:**
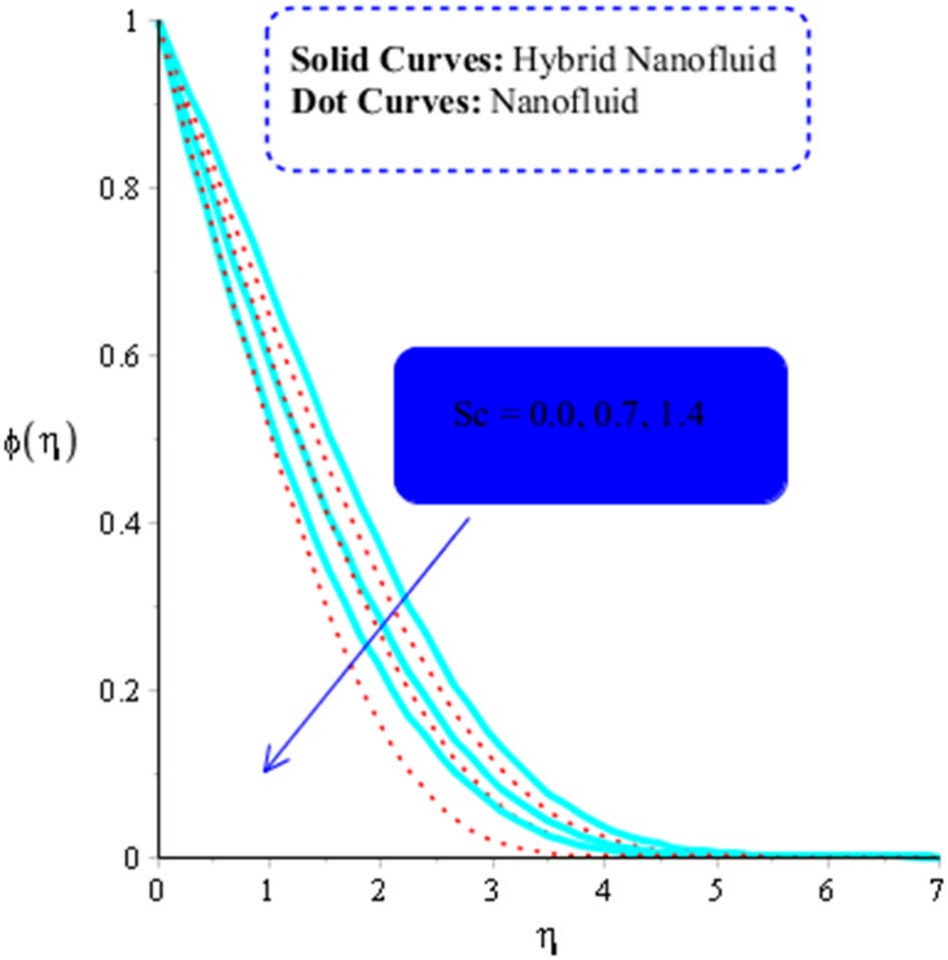
Influence of $$Sc $$ on $$\phi$$ when $$Du = 0.2, M = 0.7, Sr = 0.1, \beta = 0.2, Pr = 5,\left( {Gr} \right)_{c} = 0.3,K^{*} { } = 0.1,\beta^{*} = 0.2, Ec = 0.001, $$ and $$ \left( {Gr} \right)_{t} = 0.5, Du = 0.5, \varepsilon_{1} = 0.3, \varepsilon_{2} = 0.5, \alpha_{1} = 0.5, \alpha_{2} = 3.0.$$

The parameters $$ Sr$$, $$\left( {Gr} \right)_{c}$$, and $$Sc,$$ respectively, determine the impact of temperature gradient, Buoyancy force due to concentrations difference and diffusion coefficient on concentration field. Their influence on concentrations can be seen from Figs. 14, 15 and 16. Hence an increasing effect of $$Sr $$ and $$\left( {Gr} \right)_{c}$$ can be noticed in Figs. 14 and 15. On the other hand, concentration field decreases as a function $$Sc$$ (Fig. 16).

### Mass flux, heat transfer rate, and wall shear stresses

Investigations are conducted into the relationship between numerical data on wall stresses in the $$x and y$$ directions, wall heat transfer rate, and wall mass flow rate for both fluids, MoS_2_-fluid (mono nanofluid) and MoS_2_-Ag-fluid (hybrid nanofluid) (see Table 3). Table 3 provides an overview of the numerical results. The $$k^{*}$$ appears to be negatively correlated with the number of voids in the porous medium. As a result, the stress (or resistive force) per unit area rises. Wall shear stresses are therefore increasing functions of $$k^{*}$$ in both the x and y directions. Both the mass-flux and the temperature gradient are diminishing effects of $$k^{*}$$. Additionally, it has been found that increasing $$Du$$ causes an increase in wall shear stress. However, a surge in the wall mass transfer coefficient against $$ Du$$ is observed. Lastly, $$Sr$$ determines the temperature difference on solute particles, and an increase in $$Sr$$ causes a reduction in wall shear stress. For $$Sc$$, the opposite tendency is shown.

**Table 3:** Simulated physical quantizes when $Du=0.5, M=0.8, Sr=0.4, \beta =0.2, Pr=3,{\left(Gr\right)}_{c}=0.5,{K}^{*} =0.2,{\beta }^{*}=1.2, Ec=3, Sc=7,$ and ${\left(Gr\right)}_{t}=0.2.$

	− ${C}_{fx}{\left(Re\right)}^{1.5}$	− ${C}_{fy}{\left(Re\right)}^{1.5}$	− $Nu{\left(Re\right)}^{1.5}$	− $Sh{\left(Re\right)}^{1.5}$
k*				
0.3	0.5854736654	0.4251622865	1.469615409	1.336014008
0.7	0.5908495342	0.5872447698	1.477060316	1.342782105
0.9	0.6643955706	0.594703677	1.480258693	1.376598812
**Du**				
0.2	0.5264339688	1.137228409	1.457396180	1.324905619
0.5	0.5042650248	1.119093231	1.424234505	1.310213186
1.3	0.5000114186	1.010257923	1.417014987	1.302740897
**Sr**				
0.0	0.5000114159	1.210257919	1.778926707	1.617206098
0.7	0.4856589213	1.138660768	1.726486241	1.604078401
1.6	0.4835117384	1.108346245	1.718368259	1.525789326
**Sc**				
0.0	0.4835117388	1.238346247	2.235444588	2.032222353
0.7	0.4835117399	1.238346247	2.563934111	2.330849192
1.5	0.4835117384	1.238346245	2.805765261	2.550695691

## Core points and conclusions

The vertical 3D melting interface is used to characterize the thermal energy and mass transport characteristics that have a substantial impact on nanoparticles and hybrid nanoparticles. On a Newtonian fluid, the cumulative effects of heat transfer, a porous medium, heat gradient, rates of mass transport, and heat conduction are considered. Along with the phenomenon of heat generation, non-Furrier’s law is used in the energy equation. To determine numerical and graphical results related to velocity and temperature by different factors, G-FEA (Galerkin finite element algorithm) is used. The following is a list of the study's principal conclusions:

- Convergence study is tested observing by 300 elements;
- Approach of Hybrid nanoparticles is estimated as efficient to achieve maximum production of energy into fluidic particles as compared for nanofluid;
- The magnetic field parameter slows down particle velocity;
- As thermal energy reaches its maximum, in contrast to the given values of the Eckert number, bouncy forces, and magnetic parameter.
- Role of variable thermal conductivity number rises growth of heat energy;
- In comparison to higher values of the heat source number, the non- Fourier's results in decreased thermal dispersion and reduced heat transfer rate.
- 300 elements are needed for mesh free analysis.

Future applications of the Galerkin finite element algorithm (G-FEA) could include a range of physical and technological difficulties^11,50–59^. According to^60–70^, there have been several recent advancements that explore the importance of the research domain under consideration.

## Data Availability

This article has all the data that were created or evaluated during this investigation.
